# Cognitive deficits and CTG repeat expansion size in classical myotonic dystrophy type 1 (DM1)

**DOI:** 10.1186/1744-9081-2-16

**Published:** 2006-05-15

**Authors:** Stefan Winblad, Christopher Lindberg, Stefan Hansen

**Affiliations:** 1 Department of Psychology, Göteborg University, Göteborg, Sweden; 2 Institute of Neuroscience and Physiology, Sahlgrenska Academy, Göteborg University, Göteborg, Sweden; 3 Neuromuscular Center, Department of Neurology, Sahlgrenska University Hospital, Mölndal, Sweden; 4 Unit for Neuropsychology and Neuropsychiatry, Department of Neurology, Sahlgrenska University Hospital, Göteborg, Sweden

## Abstract

**Background:**

This study was designed to investigate cognitive abilities and their correlations with CTG repeat expansion size in classical Myotonic dystrophy type 1 (DM1), given that earlier studies have indicated cognitive deficits, possibly correlating with blood CTG repeats expansion size.

**Methods:**

A measurement of CTG repeat expansion in lymphocytes and an extensive neuropsychological examination was made in 47 patients (25 women and 22 men). Individual results in the examination were compared with normative data.

**Results:**

A substantial proportion of patients with DM1 (> 40%) scored worse in comparison to normative collectives on tests measuring executive, arithmetic, attention, speed and visuospatial abilities. We found significant correlations between longer CTG repeat expansion size and lower results on most tests associated with these abilities. Furthermore, the association between executive (frontal) deficits and DM1 were strengthened when considering both test results and correlations with CTG repeat expansion size in lymphocytes.

**Conclusion:**

This study showed deficits in several cognitive abilities when patients with DM1 are compared to normative collectives. Some of the neuropsychological tests associated with these abilities are correlated to CTG repeat expansion size in blood. These data highlight the importance of considering cognitive deficits when seeing patients with classical DM1 in clinical practice, but also the utility of using blood CTG repeat expansion size as a broad predictor of finding cognitive deficit in DM1.

## Background

Myotonic dystrophy type 1 (DM1) is the most common neuromuscular disorder with a debut in adult age. This progressive autosomal dominant multisystem disease is characterized by a variable clinical presentation, including ocular, neuromuscular, endocrine, cardiovascular, gastrointestinal and neurological abnormalities [1]. DM1 is categorized in four groups depending on age of onset and disease severity; the present paper concerns classical DM1, in which symptoms presents during the second decade or later [2]. The molecular basis has been identified in an unstable (CTG)n repeat located in the 3' untranslated region of the myotonin protein kinase (*DMPK*) gene on chromosome 19 [3]. CTG-repeat number ranges between 5 and 37 in the normal population, while in DM1 it exceeds 50, and can even increase to several thousand units [4]. How an expansion in a non-coding region of the gene cause the multisystemic features of the disease is debated, but the most reliable hypothesis postulates that the abnormal expansion in the *DMPK *gene causes a repeat expansion expressed at the RNA level, altering RNA processing, at least in part by interfering with alternative splicing of other genes [4].

As summarized by Ashizawa [5] pathology has been identified in the central nervous system, including cell loss, atrophy, focal white matter lesions and reduced cerebral blood flow in various cortical and subcortical areas of the brain. The frontal and parietal lobes are particularly affected [6]. Several neuropsychological studies have showed reduced IQ-levels in concert with deficits in executive function and visuospatial construction ability [7,8]. Abnormal scores on measures of visual or verbal memory have been reported by some authors [9] but not by others [10]. Mixed results have been found regarding verbal ability, speed and attention [6]. DM1 has also been associated with emotional- and personality disorder [11,12].

While disease severity and age of onset correlate with number of CTG repeats in lymphocytes [13] the association between cognition and CTG repeat number is more elusive. As shown in Table 1 several studies have identified correlations between cognitive ability (most prominently IQ measures) and CTG repeat expansion size, but later investigations have not confirmed these results. Discrepancies may be explained by methodological differences and by features of the examined patient group, including group-size and patient category. However, taken together evidence exist to conclude that blood CTG repeat expansion size correlates with cognition, especially when considering the entire DM1 spectrum, which ranges from mental retardation (congenital DM1) to subtle memory deficits (minimal DM1) [14,9]. In order to further explore the association we investigated 47 patients with classical DM1, using a comprehensive neuropsychological examination, and correlated test results with CTG repeat expansion size in blood lymphocytes.

**Table 1:** Studies investigating the correlation between neuropsychological test scores and CTG repeat expansion size in DM1.

Authors*	Number of patients	Tests	Main conclusion
Turnpenny et al. [34]	55	WAIS (short version).	Increase in repeat size associated with lower IQ.
Damian et al. [10]	28	Neuropsychological test battery^†^.	Large repeat size (>1000) associated with cognitive impairment.
Jaspert et al. [33]	14	MMSE, MWT-B, Progressive matrices.	Large repeat size (> 1000) associated with cognitive impairment.
Rubinsztein et al. [9]	36	MMSE, NART, Progressive matrices.	Mild DM1 associated with impaired memory function.
Perini et al. [25]	17	WAIS	CTG correlates with total IQ and non-verbal IQ.
Marchini et al. [13]	24	WAIS	CTG correlates with all IQ measures and short-term memory (Digit span).
Steyaert et al. [14]	24	WISC	CTG correlates with IQ in childhood type of DM1.
Meola et al. [8]	19	Neuropsychological test battery.^†^	No correlation between CTG repeat size and test performance.
Modoni et al. [24]	70	Neuropsychological test battery.^†^	No correlation between CTG repeat size and test performance.

* Referred articles are presented in the reference section.

^† ^Batteries including tests aimed to measure a wide variety of neuropsychological functions. WAIS = Wechsler Adult Intelligence Scale; MMSE = Mini Mental State Examination; MWT-B = Mehrfach- Wahl-Wortschatz-Test; NART = National Adult Reading Test; WISC = Wechslers Adult Intelligence Scale for Children.

## Methods

### Participants

Patients with DM1 were recruited from the Neuromuscular Center at the Sahlgrenska University Hospital, Mölndal, Sweden. Inclusion criteria were: age 18–65 years with no history of major psychiatric or somatic illness, acquired brain injury or alcohol misuse. Patients with the congenital, childhood or mild DM1 were excluded [2]. The diagnosis was confirmed by an expansion of CTG-repeats. Fifty-nine patients at the clinic, meeting the criteria of classical DM1 were invited to participate. Twelve declined, leaving 47 (25 women and 22 men, mean age 42 years: age range 23–62 years). Clinical and demographic background data are presented in Table 2. The range of CTG triplets repeat expansion was 70–1550. All participants gave informed consent and the medical ethics committee at the Sahlgrenska Academy, Göteborg University, approved the study. Clinical data was collected by a self-rating procedure designed at the Neuromuscular Center aimed to explore the presence or absence of different symptoms. Muscle function was measured by a physiotherapist using Brooke's grading system of mobility [15]. Furthermore an occupational therapist measured grip force using the Grippit instrument [16].

**Table 2:** Demographic and clinical description of DM1 patients

Variable	DM1 (n = 47)
Age (years)	41.55 (10.4)
Gender	22 M, 25 F
Age at onset*	27.6 (11.0, 12–50)
Education (years)	10.8 (2.1)
Married/cohabiting	60 %
Hearing impairment	28%
Vision impairment	49%
Fatigue	79 %
Day-time sleepiness	66 %
Reduced initiative	43 %
Brooke-rating**	0.51 (1.1, 0–5)
Grip force^† ^(N)	97.5 (48.5)

* Results are presented in mean (sd, range).

** Brooke's functional test and grading system measuring mobility (hips and legs). In the grading system, zero implies normal function and higher values indicates increasing levels of immobility.

^† ^Measurement of grip force using the Grippit instrument. Mean (sd) for healthy controls = 331 (77) [16].

### Neuropsychological tests

All subjects participated in a neuropsychological investigation comprising the Wechsler Adult Intelligence Scale – Revised (WAIS-R) [16] and tests measuring verbal fluency: FAS [18], visual construction and memory ability: Rey Complex Figure Test (RCFT) [19], verbal memory: Rey Auditory Verbal Learning Test (RAVLT) [20], speed: Trail Making Test A [21], Stroop Colour Word Test B (CWT B) [22], attention: Trail Making Test [21] and executive function: Wisconsin Card Sorting Test (WCST) [23], TMT B [21] and Stroop Colour Word Test B [22]. The tests were administered in a quiet room in two sessions of approximately two hours each. Following recommendations by Mitrushina et al. [22] individual performance was compared to age-normative data (see reference for respective test above).

### Genetic analysis

DNA was extracted from peripheral blood lymphocytes and analysed for expansion of the CTG-repeat in the DMPK gene. The analysis was performed with PCR and southern blot using the probe pM10M6 [3]. The size of CTG-expansion was assessed visually from exposed x-ray films. As most patients with DM1 show a smear rather than a distinct band, because of somatic mosaicism, the approximate midpoint of the smear was reported.

### Statistical analysis

Data were analysed using SPSS base 11.5 (Chicago, IL) and are presented as median and interquartile range except from demographic and clinical data. The Spearman rank correlation test was used to analyse correlations between CTG repeat number and scores on separate neuropsychological tests. The significance level was set at 0.05 for all parameters.

## Results

**Figure 1:**
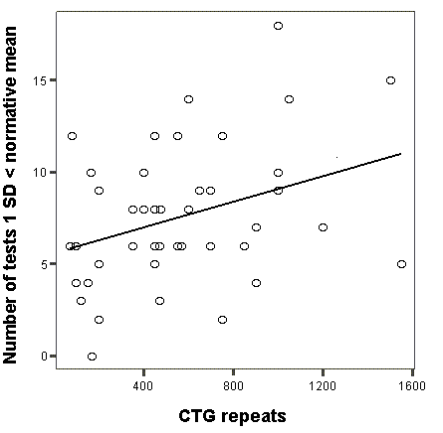
**Correlation between CTG repeats expansion size and total number of tests 1 SD < normative mean**. Correlation between CTG repeats expansion size and total number of tests 1 SD < normative mean in patients with DM1 (P = < 0.05, rho = 0.30).

The results of test performance and correlations with CTG-repeat expansion are presented in Table 3. Mean total IQ (WAIS-R) was 95. As compared to normative data, and using 1 SD below normative mean as a sign of impairment, patients with DM1 scored worse on a majority of tests (in the normative collectives 16 % of subjects are expected to perform 1 SD below mean). More than 40 % of the patients scored 1 SD < normative mean on tests associated with executive function (WCST, TMT B, CWT B), speed (TMT, Digit symbol, CWT B), attention (Digit span, Digit symbol, TMT), visuospatial- (RCFT, Object assembly, Block design) and arithmetic ability (Arithmetic). Furthermore, patients with DM1 scored worse than normative samples on RAVLT (immediate recall), Picture Arrangement, RCFT (delayed recall), Vocabulary and Similarities. On average, DM1 patients scored 1 SD below the mean on 7 of 20 tests (median = 7, range = 0 -18) in the examination. Most constituent tests (except from WCST and RAVLT) correlated positively with one another. No combination of aberrant tests (defined as 1 SD < normative mean) was found to be typical for the group of DM1 patients. Rather the pattern of combinations was heterogeneous. To explore if any of the tests in the data set could predict total number of test failed we investigated the correlation between results on each individual test and the total number of tests failed. We found significant correlations between a majority of tests (mean Rho = -0.539) and the total number of failed tests for each patient, indicating that no test had unique predictive value. Significant negative correlations with CTG-repeat expansion size were found on tests associated with executive function (WCST, CWT B and TMT B), arithmetic (Arithmetic), visuospatial construction (RCFT, copy trial), speed and attention (TMT) and verbal ability (Vocabulary and Comprehension). In addition, as illustrated in Figure 1, the number of CTG triplets correlated with global cognitive impairment (i.e. number of tests with results 1 SD < normative mean). Patients reporting subjective problems with fatigue, hearing and/or vision were undistinguishable from the patients without such problems, as assessed with the Wilcoxon rank sign test. There were no gender differences in the performance on the neuropsychological tests in the DM1 group.

**Table 3:** Performance on neuropsychological tests and correlationwith CTG repeat expansion*

Test	DM1 (n = 47)	1 SD < mean**	2 SD < mean^†^	Spearman's Rho
FAS	35.0 (15.0)	34.0 %	2.1 %	-.02
RCFT (copy)	31.0 (8.0)	44.7 %	36 %	-.34 ^†^
RCFT (delayed recall)	16.5 (10.5)	23.4 %	21.3 %	-.09
Stroop CWT B (seconds)	158.0 (54.0)	57.5 %	27.2 %	-.46^†^
RAVLT (immediate recall)	6.0 (9.0)	34.0 %	8.5 %	-.10
RAVLT (delayed recall)	11.0 (4.0)	10.6%	0 %	.19
WCST (categories)	3.0 (4.0)	70.2 %	17 %	-.38^†^
TMT A (seconds)	42.0 (20.0)	61.7 %	25.8 %	-.39^†^
TMT B (seconds)	101.0 (54.0)	87.2 %	63.8 %	-.30^†^
**WAIS-R:**				
Verbal subtests:				
Information	21.0 (8.0)	6.4 %	0 %	.08
Digit span	12.0 (4.0)	44.7 %	8.5 %	-.03
Vocabulary	45.0 (14.0)	19.1 %	0 %	-.34 ^†^
Arithmetic	9.0 (4.0)	40.5 %	4.3 %	-.41 ^†^
Comprehension	23.0 (6.0)	8.5 %	0 %	-.30 ^†^
Similarities	19.0 (6.0)	17 %	0 %	-.09
Nonverbal subtests:				
Picture completion	16.0 (3.0)	10.6 %	0 %	-.08
Picture arrangement	11.0 (7.0)	25.5 %	0 %	-.21
Block design	20.0 (11.0)	42.5 %	2.1 %	-.09
Object assembly	23.0 (15.0)	53.2 %	10.6 %	.04
Digit symbol	40.0 (16.0)	57.5 %	4.3 %	-.23

* Results are presented in raw score (median and interquartile range).

** DM1 patients scoring 1 SD < normative mean.

^† ^DM1 patients scoring 2 SD < normative mean.

^‡ ^Significant correlation (< 0.05) between test performance and CTG repeat expansion in blood lymphocytes.

RCFT = Rey Complex Figure Test; CWT = Colour Word Test; RAVLT = Rey Auditory Verbal Learning Test; WCST = Wisconsin Card Sorting Test; TMT = Trail Making Test.

## Discussion

This study showed a significant negative correlation between CTG repeat expansion size and scores on tests depending on executive, arithmetic, attention, visuospatial, speed and verbal ability. This means that poorer performance on these tests is associated with longer CTG-repeats as measured in blood lymphocytes (indicating an association with repeat size). Furthermore, a substantial proportion of DM1 patients scored worse on tests associated with these abilities (verbal ability excluded). Differences in neuropsychological performance between patients with classical DM1 and controls have been shown in earlier studies on tests measuring speed and attention [25], executive [8], visuospatial [7] and arithmetic ability [25]. Our study not only confirms these results but also show significant correlations between poorer results on certain tests associated with these abilities and larger CTG repeat expansion size as measured in blood. Note, however, that certain tests associated with speed and attention (Digit span, Digit symbol) and visuospatial construction ability (Block design and Object assembly) are *not *correlated with CTG expansion size, making this association more uncertain. Furthermore, poor test performance, as defined here, indicates that the result is in the borderline-range and not necessarily associated with severe cognitive impairment [22]. However, results on TMT B seem particularly problematic for DM1 patients, in that 64 % of the patients score 2 SD < mean (indicating severe impairment). Besides being an indicator of executive dysfunction, TMT B is associated with several cognitive functions and, correspondingly, diffuse brain damage [21].

Two forms of intelligence have been distinguished: fluid intelligence (FI) (abilities to acquire new concepts and to adapt to unfamiliar situations) and crystallized intelligence (CI) (a system of well-learned intellectual skills or knowledge accumulated over a lifetime) [26]. The acquisition of knowledge (CI) depends on well-functioning attention, speed and problem-solving ability (FI). When reviewing the cognitive profile in DM1 a picture emerges that connects to these aspects of intelligence. Results broadly indicate performance in the normal range on tests associated with CI (verbal subtests/semantic knowledge in WAIS-R) and below the normal range on tests measuring FI (nonverbal subtests in WAIS-R and tests depending on speed and executive ability). Given that CI depends upon the integrity of FI, one might infer that FI-related dysfunctions arise late in classical DM1; early deficits would be expected to manifest themselves as deficits also in CI. The FI-CI pattern in DM1 also broadly resembles the cognitive profile associated with aging [27]. In this context, it is interesting to note that DM1 has been termed a progeroid (early aging) syndrome [28], with a premature decline of age somatic/brain functions [5]. However, cognitive decline have been reported by some authors [29] but not by others [30]. Taking the progeroid nature of DM1 into account, it is reasonable to perform further longitudinal studies on cognition in classical DM1.

The presence of somatic mosaicism in the tissues of patients with DM1 has been noted in earlier studies [31] and challenges the assumption that CTG repeat expansion as measured in blood lymphocytes correspond to CTG repeat expansion in brain (and indirectly cognition). Sergeant et al. [32] have documented the presence of very large CTG expansions in brain tissue of DM1 patients characterized by much smaller size of CTG in lymphocytes. These authors also documented mosaicism in different brain areas. Modoni et al. [24] used mosaicicm to explain the absence of correlation between blood CTG repeats and neuropsychological test performance. Our data did reveal a significant correlation between blood CTG and certain aspects of cognition and this suggests at least some value of using CTG repeat expansion size in lymphocytes as a broad predictor of cognitive impairment in DM1. This estimate should however be used with caution when considering the heterogeneity that actually appear when examining the number of cognitive deficits shown by patients with similar CTG-repeat expansion sizes (Figure 1).

The significance of other impairments, such as reduced eyesight and daytime fatigue, on neuropsychological functioning is uncertain [33]. One drawback of our and other studies, as summarized in Ashizawa [5] and D'Angelo and Bresolin [6], is that they do not assess these features in sufficient detail to permit evaluation of their significance. When considering CTG repeat expansion as measured in blood, one might speculate that this measure at the most indicates a *general *disease process, due to the fact that CTG-repeat expansion affect different bodily tissues [13]. If true, the correlation between CTG repeat size and cognition may not only be influenced by brain-related pathology [10] but also by fatigue, poor eyesight and manual weakness. These potential predictors should be explored in future studies on the cause of cognitive deficits in DM1.

## Conclusion

This study show deficits in tests associated with executive, visuospatial, arithmetic, attention and speed dependent ability when patients with DM1 are compared to normative collectives. Some of the neuropsychological tests associated with these abilities are correlated to CTG repeat expansion size in blood. These data highlight the importance of considering cognitive deficits when seeing patients with classical DM1 in clinical practice, but also the utility of using blood CTG repeat expansion size as a broad predictor of finding cognitive deficit in classical DM1.

## Competing interests

The author(s) declare that they have no competing interests.

## Authors' contributions

SW carried out the neuropsychological examination and participated in the conception, design, analyses, interpretation and writing of the study. CL participated in the design, acquisition of data and helped to draft the manuscript. SH participated in the design, statistical analyses and drafted the manuscript. All authors read and approved the final manuscript.
